# An Interpretable Machine Learning Model Based on Inflammatory–Nutritional Biomarkers for Predicting Metachronous Liver Metastases After Colorectal Cancer Surgery

**DOI:** 10.3390/biomedicines13071706

**Published:** 2025-07-12

**Authors:** Hao Zhu, Danyang Shen, Xiaojie Gan, Ding Sun

**Affiliations:** Department of General Surgery, The First Affiliated Hospital of Soochow University, Suzhou 215005, China; 20235232130@stu.suda.edu.cn (H.Z.); shendanyang@suda.edu.cn (D.S.)

**Keywords:** prediction model, machine learning, metachronous liver metastasis, colorectal cancer, CRC

## Abstract

**Objective**: Tumor progression is regulated by systemic immune status, nutritional metabolism, and the inflammatory microenvironment. This study aims to investigate inflammatory–nutritional biomarkers associated with metachronous liver metastasis (MLM) in colorectal cancer (CRC) and develop a machine learning model for accurate prediction. **Methods**: This study enrolled 680 patients with CRC who underwent curative resection, randomly allocated into a training set (n = 477) and a validation set (n = 203) in a 7:3 ratio. Feature selection was performed using Boruta and Lasso algorithms, identifying nine core prognostic factors through variable intersection. Seven machine learning (ML) models were constructed using the training set, with the optimal predictive model selected based on comprehensive evaluation metrics. An interactive visualization tool was developed to interpret the dynamic impact of key features on individual predictions. The partial dependence plots (PDPs) revealed a potential dose–response relationship between inflammatory–nutritional markers and MLM risk. **Results**: Among 680 patients with CRC, the cumulative incidence of MLM at 6 months postoperatively was 39.1%. Multimodal feature selection identified nine key predictors, including the N stage, vascular invasion, carcinoembryonic antigen (CEA), systemic immune–inflammation index (SII), albumin–bilirubin index (ALBI), differentiation grade, prognostic nutritional index (PNI), fatty liver, and T stage. The gradient boosting machine (GBM) demonstrated the best overall performance (AUROC: 0.916, sensitivity: 0.772, specificity: 0.871). The generalized additive model (GAM)-fitted SHAP analysis established, for the first time, risk thresholds for four continuous variables (CEA > 8.14 μg/L, PNI < 44.46, SII > 856.36, ALBI > −2.67), confirming their significant association with MLM development. **Conclusions**: This study developed a GBM model incorporating inflammatory-nutritional biomarkers and clinical features to accurately predict MLM in colorectal cancer. Integrated with dynamic visualization tools, the model enables real-time risk stratification via a freely accessible web calculator, guiding individualized surveillance planning and optimizing clinical decision-making for precision postoperative care.

## 1. Introduction

Colorectal cancer (CRC), a prevalent gastrointestinal malignancy worldwide, ranks as the second leading cause of cancer-related mortality [1]. The liver’s unique immune-tolerant microenvironment enables CRC cells to evade immune surveillance and establish metastatic foci, resulting in a high propensity for hepatic metastasis [2]. Studies demonstrate that 30–50% of patients with CRC develop liver metastases, with MLM occurring in 15–25% of postoperative cases [3,4]. Although surgery combined with systemic therapy partially improves outcomes, the rates of recurrence and new metastases remain substantial, with 5-year survival below 30% [5]. Immune dysregulation, systemic inflammation, and nutritional abnormalities exhibit specific associations with tumorigenesis and progression [6,7]. Inflammatory–nutritional biomarkers have emerged as crucial prognostic indicators for various malignancies by reflecting systemic inflammatory status and nutritional conditions [8,9,10,11]. In gastric cancer, both the SII and PNI independently predict postoperative outcomes, while their combination significantly enhances prognostic accuracy for recurrence risk and survival [9]. Therefore, developing a multiparameter predictive model incorporating systemic inflammatory–nutritional biomarkers may enable early and precise prediction of metachronous liver metastasis after CRC surgery, offering significant clinical translational value.

Although substantial research has been conducted on postoperative metastasis prediction, existing conventional prediction models are subject to several important limitations. The clinical feature-integrated prediction model developed by Lu et al., while valuable, exhibits several areas requiring improvement: first, the modest sample size (n = 161, including only 59 MLM events) may limit the model’s robustness; second, the sole reliance on traditional linear regression not only prevents comprehensive algorithmic comparisons but also fails to adequately capture the complex nonlinear relationships among MLM risk factors; and third, the lack of web-based implementation significantly hinders clinical adoption [12]. These limitations collectively reduce the model’s clinical utility. In this context, machine learning (ML) approaches have emerged as powerful tools for cancer prognosis prediction, demonstrating superior performance through the analysis of large-scale clinical datasets [13]. Considerable progress has been made in applying ML models to predict colorectal cancer liver metastases, with high predictive accuracy achieved in particular for synchronous liver metastasis (SLM). However, current models remain predominantly focused on SLM prediction, with no dedicated framework available for predicting MLM after curative resection. Moreover, a critical gap exists in the incorporation of key biomarkers reflecting systemic inflammatory status and nutritional metabolism [14,15]. Despite the strong predictive performance of ML models, their inherent “black-box” nature—specifically, the lack of transparency regarding individual feature contributions—poses a major barrier to clinical translation [16]. To overcome this challenge, we implemented SHAP to quantify feature importance and provide visual interpretation of model decisions, thereby improving clinical interpretability [17]. Furthermore, we applied GAMs to fit SHAP values, revealing nonlinear relationships between risk factors and outcomes while identifying critical thresholds for MLM risk stratification. To the best of our knowledge, this study represents the first application of this methodology for determining optimal cutoff values of MLM-associated risk factors.

This study aims to develop a machine learning-based predictive tool for postoperative MLM in patients with CRC. Through systematic comparison of predictive performance across different algorithms, we established a high-performance prediction model deployed via an online platform. This implementation will enable clinically accessible personalized risk assessment, thereby advancing precision medicine in postoperative management.

## 2. Materials and Methods

### 2.1. Study Population

This study retrospectively included consecutive cases treated at our hospital from January 2017 to June 2023. Inclusion criteria: (1) histologically confirmed primary colorectal cancer; (2) complete preoperative baseline data with regular postoperative follow-up ≥6 months; (3) no history of other malignancies at initial treatment; (4) receipt of standard radical resection with guideline-recommended adjuvant therapy. Exclusion criteria: (1) history of primary/metastatic liver tumors; (2) perioperative mortality or loss to follow-up; (3) receipt of neoadjuvant chemotherapy or radiotherapy; (4) presence of severe cardiovascular or cerebrovascular diseases that could significantly impact prognosis. This study was approved by the Ethics Committee of the First Affiliated Hospital of Soochow University (2024513).

### 2.2. Data Collection

Clinical data of patients with CRC were extracted from the HAITAI electronic medical record system (version 4.0), including demographic characteristics (sex, age, BMI), tumor characteristics (tumor size, T stage, N stage, differentiation grade, vascular invasion, perineural invasion, maximal size of regional lymph node), comorbidities and surgical details (diabetes, fatty liver, colonic obstruction, operation time, intraoperative blood loss), and the prognostic nutritional index (PNI), calculated as PNI = serum albumin (g/L) + 5 × lymphocyte count (10^9^/L). Inflammatory status was assessed using two indices: (1) the systemic immune–inflammation index (SII), determined by platelet count × neutrophil-to-lymphocyte ratio, and (2) the systemic inflammation response index (SIRI), calculated as neutrophil count × monocyte-to-lymphocyte ratio. Liver function was evaluated using the albumin–bilirubin (ALBI) index, computed as [−0.085 × albumin (g/L)] + [0.66 × log_10_ bilirubin (μmol/L)]. The modified Glasgow Prognostic Score (mGPS) was graded as follows: Grade 0 (albumin ≥ 35 g/L and C-reactive protein ≤ 10 mg/L), Grade 1 (albumin < 35 g/L or C-reactive protein > 10 mg/L), and Grade 2 (albumin < 35 g/L and C-reactive protein > 10 mg/L). All blood markers were based on results obtained within one week prior to surgery.

### 2.3. Research Methods

We implemented a rigorous hybrid feature selection approach combining the Boruta algorithm and Lasso regression to optimize both predictive performance and clinical interpretability. The Boruta algorithm was employed as an all-relevant-feature selector that iteratively evaluates variable importance against randomized shadow features through random forest modeling, effectively identifying stable predictors with significance exceeding noise levels (*p* < 0.01) while capturing complex nonlinear relationships characteristic of biological systems [18]. This was complemented by Lasso regression with λ determined through 10-fold cross-validation, which provided essential regularization to eliminate collinear features and generate a sparse, interpretable model suitable for clinical implementation. The intersection of features selected by both methods ensured robust predictor identification while maintaining model simplicity. Parameter selection was based on empirical evidence showing that 500 trees and iterations achieve stable feature importance rankings in Boruta, while α = 1 in Lasso optimally balances feature selection and prediction accuracy for clinical datasets.

**Figure 1:**
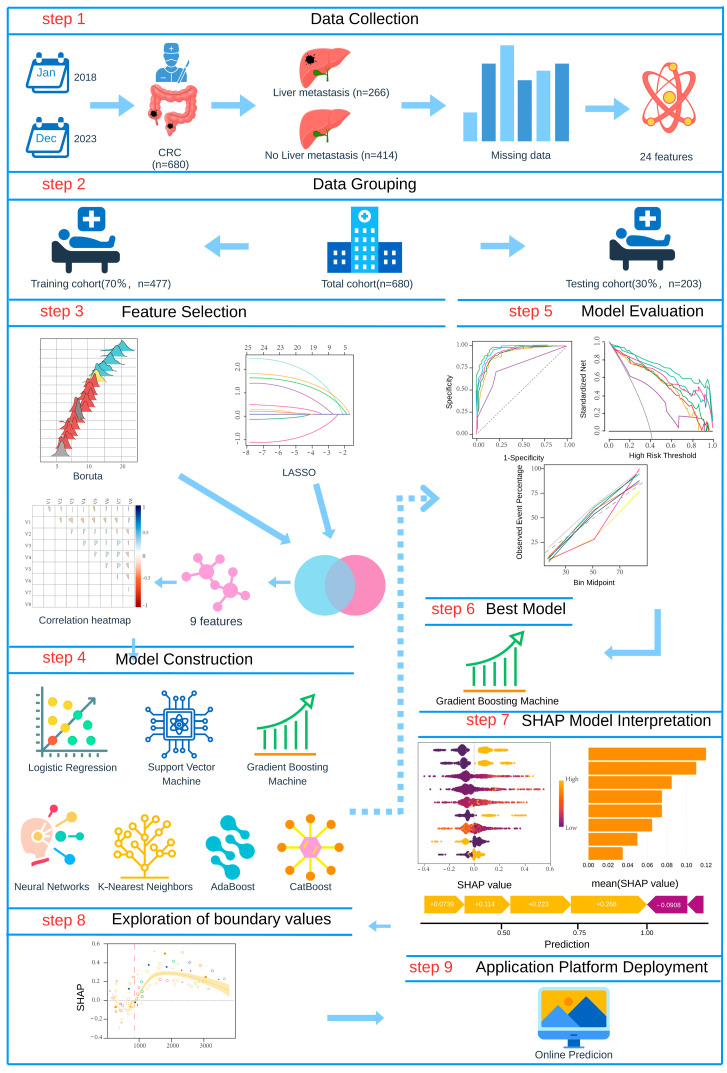
Flow diagram of the predictive model.

Subsequently, we constructed a predictive modeling framework incorporating seven machine learning algorithms: logistic regression (LR), support vector machine (SVM), gradient boosting machine (GBM), neural networks, K-nearest neighbors (KNN), AdaBoost, and CatBoost. We systematically evaluated each model’s performance using receiver operating characteristic (ROC) curves, decision curve analysis (DCA), and calibration curves across training and validation sets, along with comprehensive comparisons of accuracy, sensitivity, specificity, precision, and F1-score, ultimately identifying the optimal predictive model. For model interpretability analysis, we employed the SHAP analysis to generate summary plots and feature importance bar charts, elucidating the global contribution of each feature to the predictive outcomes. For continuous variables, we applied GAMs to nonlinearly model SHAP values, identifying critical thresholds where features exerted directional effects on predictions by analyzing the intersection points between GAM-fitted curves and the zero baseline (SHAP = 0). A transition from negative to positive SHAP values indicated a shift toward a positive predictive effect, whereas the reverse suggested a suppressive influence. Finally, we developed a clinically applicable predictive platform, establishing a complete research pipeline from feature selection and model construction to clinical translation. Figure 1 illustrates the comprehensive workflow of this study.

### 2.4. Data Analysis

This study conducted statistical description and intergroup comparative analysis of patients’ demographic characteristics and clinical parameters. For categorical variables, either the chi-square test or Fisher’s exact test was employed for intergroup comparisons based on data characteristics, with results presented as counts (percentages). Continuous variables were described using the mean (standard deviation), with the independent samples *t*-test used for intergroup comparisons. All statistical analyses were performed using R version 4.4.3.

## 3. Results

### 3.1. Patient Characteristics

This retrospective study enrolled patients with colorectal cancer who underwent radical surgery at the First Affiliated Hospital of Soochow University between January 2018 and December 2023. After screening and excluding cases that did not meet the study criteria, 680 patients with colorectal cancer were ultimately included, among whom 266 (39.1%) developed liver metastases at least 6 months postoperatively. The research team systematically collected 25 clinical parameters, with 6 variables (fatty liver [1.7%], N stage [2.9%], colonic obstruction [3.7%], maximal size of regional lymph node [2.6%], blood loss [1.8%], and tumor size [1.2%]) exhibiting minor data missingness as illustrated in Figure S1. Missing continuous variables were imputed using mean values, while categorical variables were imputed with mode values [19]; all other parameters were complete without missing data. Table 1 presents the baseline demographic and clinicopathological characteristics of the entire cohort and its stratified training and validation cohorts, whereas Table 2 delineates the comparative clinical profiles between CRC and MLM in the training cohort.

### 3.2. Feature Selection

**Figure 2:**
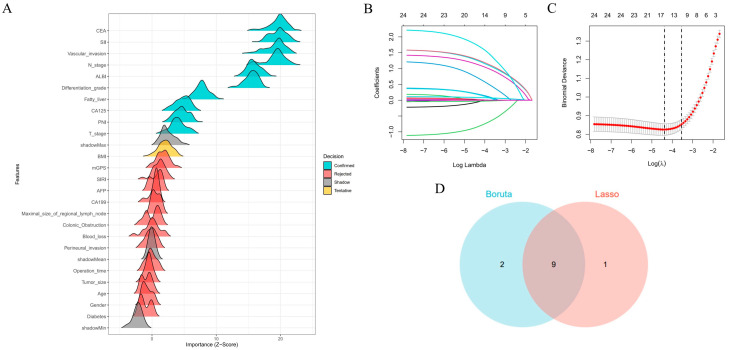
Feature selection process and consensus predictor identification: (**A**) Boruta algorithm-based feature selection categorized predictors as important (blue), tentative (yellow), or rejected (red). (**B**) LASSO coefficient trajectories across regularization penalties. (**C**) Cross-validation error curve for optimal λ selection. (**D**) Venn diagram of overlapping predictors from Boruta and LASSO, identifying nine consensus features for modeling.

**Figure 3:**
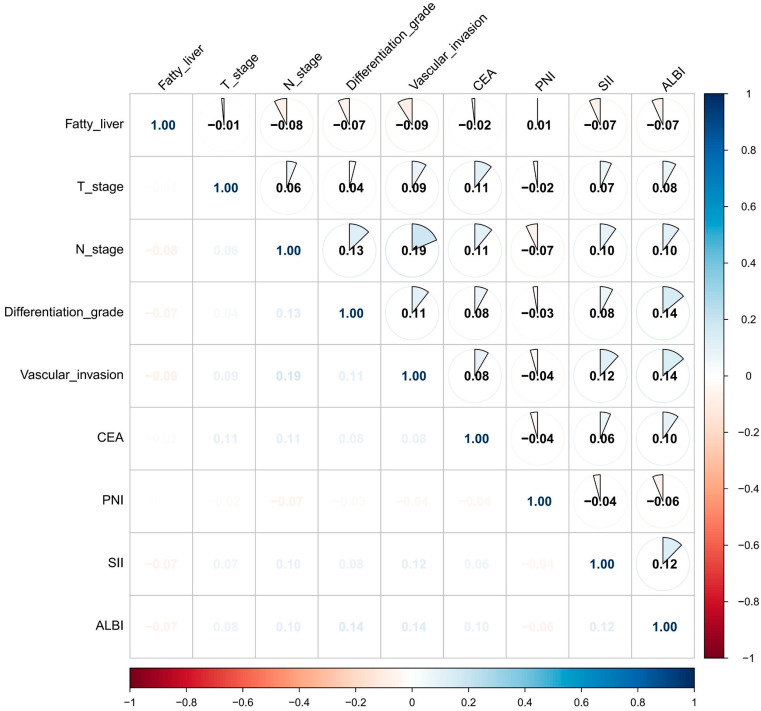
Heatmap of the correlation among the nine variables used for model construction.

We implemented Boruta feature selection (significance threshold *p* = 0.01, 100 iterations) to classify features into three distinct categories: significant predictors were defined as variables demonstrating consistently and statistically significantly higher importance than the maximum importance of all shadow features; tentative predictors represented variables with statistically non-significant yet borderline importance, characterized by importance distributions fluctuating around the shadow feature threshold and warranting further validation; and rejected predictors exhibited persistently lower importance than the shadow feature distribution. The analysis conclusively identified fatty liver, T stage, N stage, differentiation grade, vascular invasion, CA125, CEA, PNI, SII, and ALBI score as significant predictors (blue markers). BMI was classified as a tentative predictor (yellow markers) due to its unstable importance relative to the shadow feature threshold, with all remaining variables rejected (red markers, Z-scores below shadow region) (Figure 2A). LASSO regression demonstrated distinct feature preferences, excluding BMI and CA125 identified by Boruta while additionally recognizing maximal size of regional lymph node as a predictive feature (Figure 2B,C). The intersection of both methods yielded nine consensus predictors—fatty liver, T stage, N stage, differentiation grade, vascular invasion, CEA, PNI, SII, and ALBI score (Figure 2D)—which were subsequently used for model construction. To assess potential multicollinearity among selected features, a diagnostic correlation heatmap was generated to evaluate inter-feature relationships. Figure 3 demonstrates that all pairwise correlation coefficients between final predictors were below 0.2, confirming satisfactory feature independence.

### 3.3. Constructing and Validating Models

**Figure 4:**
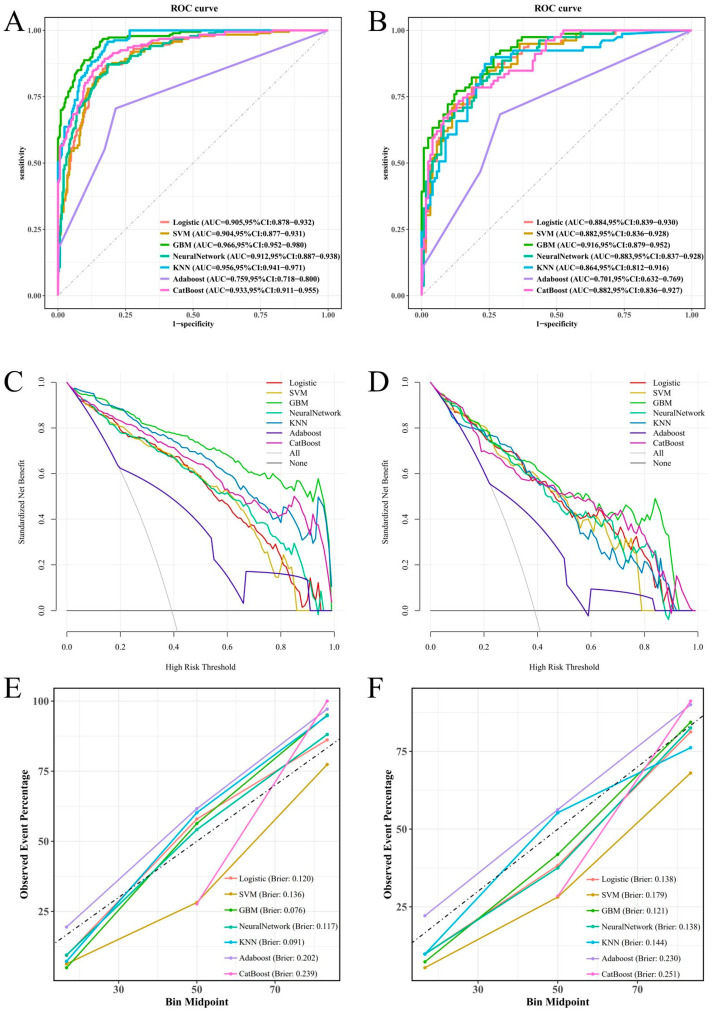
To assess the predictive ability of the seven machine learning models in the training and validation cohorts, we utilized various evaluation tools including the ROC curve (**A**,**B**), DCA plot (**C**,**D**), and calibration plot (**E**,**F**).

**Figure 5:**
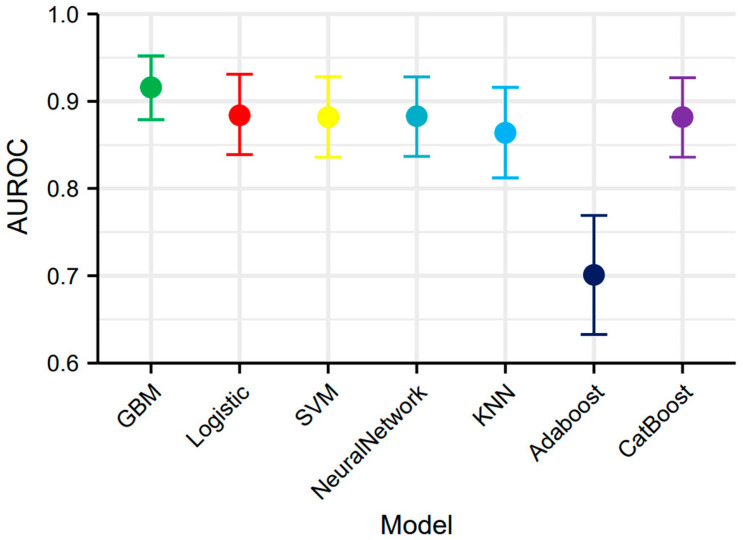
AUROC with 95% confidence intervals of seven machine learning models.

We developed seven machine learning models (logistic regression, SVM, GBM, ANN, KNN, AdaBoost, and CatBoost) using the selected feature variables. The parameters of each model are presented in Table S1. ROC curve analysis across the training and validation sets revealed distinct discriminative performances among the models. The GBM model demonstrated superior discriminative ability in the validation set, achieving an area under the curve (AUC) of 0.916 (95% CI: 0.879–0.952), significantly outperforming other models. Logistic regression (AUC = 0.884), SVM (AUC = 0.882), and CatBoost (AUC = 0.882) also showed satisfactory performance, while AdaBoost (AUC = 0.701) exhibited relatively poor discriminative ability (Figure 4A,B). The DCA curve indicated that the GBM model provided the highest clinical net benefit across most clinically relevant threshold probabilities (Figure 4C,D). Calibration curve analysis further confirmed that the GBM model achieved optimal agreement between predicted probabilities and observed frequencies, with the lowest Brier score (0.121), indicating superior calibration (Figure 4E,F). Based on its significantly superior AUROC in the validation set (Figure 5), GBM was established as the final model. This model maintained robust performance across other metrics (accuracy, sensitivity, specificity, precision, F1-score), with complete validation-set performance detailed in Table 3 and training-set metrics provided in Table S2.

### 3.4. Feature Contribution Analysis via SHAP Values

**Figure 6:**
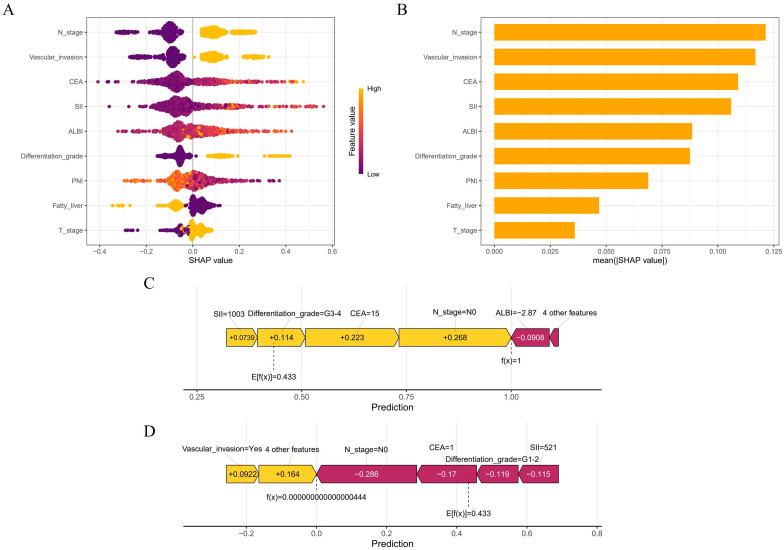
The Shapley Additive Explanations method was used to interpret the GBM model. (**A**) Summary plot showing feature impact directionality. (**B**) Feature importance ranking by mean absolute SHAP values. (**C**,**D**) Force plots illustrating individual prediction explanations for representative MLM and non-MLM cases.

**Figure 7:**
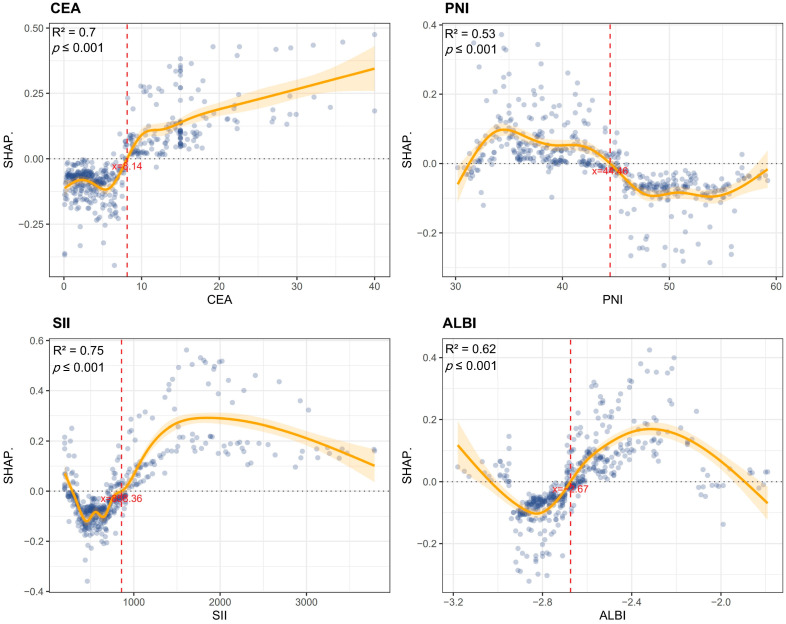
Nonlinear predictor effects on MLM risk. Partial dependence plots identified critical thresholds: CEA > 8.14 ng/mL, SII > 853.36, and ALBI > −2.67 marked increased risk, while PNI > 44.46 showed protection.

This study employed SHAP analysis to elucidate the contribution patterns of features in the GBM model for predicting colorectal cancer MLM. The SHAP summary plot visually demonstrates both the direction and magnitude of feature contributions, where positive values indicate increased metastasis risk and negative values denote protective effects. N stage and vascular invasion exhibited the strongest positive predictive effects, while elevated CEA and SII also showed significant positive associations with MLM risk. Conversely, ALBI score predominantly demonstrated negative SHAP values, suggesting that preserved liver function confers protection (Figure 6A). The SHAP bar chart ranked by mean absolute SHAP values confirmed the paramount predictive role of N stage and vascular invasion, whereas fatty liver and T stage showed relatively modest effects (Figure 6B). We further conducted case-specific SHAP value decomposition for two representative cases using the GBM algorithm. In an MLM-positive case, the combined effects of an advanced N stage (N1–2), elevated CEA (15 ng/mL), poor differentiation (G3–4), and high SII (1003) collectively increased the prediction score from baseline (E[f(x)] = 0.433) to a final value of 1 (Figure 6C). In contrast, an MLM-negative case showed protective effects from an N0 stage, low CEA (1 ng/mL), well-differentiated tumor (G1–2), and moderate SII (521), despite positive contributions from vascular invasion (+0.0922) and four other features (+0.164), resulting in a final prediction below baseline (Figure 6D). PDPs revealed complex nonlinear relationships between predictors and outcomes. GAMs were fitted to quantify threshold effects of the CEA, PNI, SII, and ALBI on MLM risk by modeling their relationships with SHAP values. The models demonstrated good goodness of fit (CEA-R^2^ = 0.70, PNI-R^2^ = 0.53, SII-R^2^ = 0.75, ALBI-R^2^ = 0.62). All variables showed statistically significant nonlinear associations with MLM risk (*p* < 0.001). Specifically, MLM risk increased with rising CEA levels, with SHAP values transitioning from negative to positive at CEA > 8.14 ng/mL, suggesting that this threshold may indicate enhanced tumor aggressiveness. Similarly, SII > 853.36 marked a positive transition in SHAP values, reflecting the pro-metastatic effect of systemic inflammation. For ALBI score, a statistically significant directional change occurred at >−2.67, indicating a potential threshold for liver function-associated risk alteration. PNI > 44.46 exhibited protective effects, confirming that better nutritional status reduces MLM risk (Figure 7).

**Figure 8:**
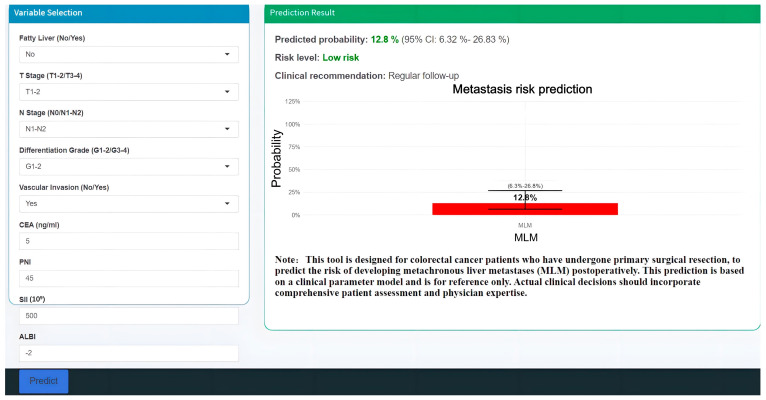
Clinical deployment of the GBM prediction model as an internet-accessible tool for MLM.

To facilitate clinical translation, we developed an interactive web-based calculator implementing our predictive model (https://haozhu-online-app.shinyapps.io/make/, accessed on 30 June 2025), enabling real-time risk assessment at the point of care (Figure 8).

## 4. Discussion

Hepatic metastasis is strongly associated with significantly reduced overall survival in patients with CRC. Accurate prediction of postoperative MLM risk is critical for formulating personalized adjuvant treatment strategies. Recent studies have validated the prognostic value of inflammatory–nutritional biomarkers in malignancies: a decreased PNI significantly correlates with aggressive progression and poor outcomes in gastric cancer [9], while an elevated SII serves as an independent risk factor for recurrence and survival post-hepatectomy [10]. However, despite their demonstrated predictive potential in individual cancer types, these biomarkers exhibit limited efficacy when used independently, failing to meet the clinical demand for precise risk stratification. Notably, while previous studies have successfully incorporated multidimensional nutritional–inflammatory biomarkers into prognostic models for gallbladder cancer and intrahepatic cholangiocarcinoma with satisfactory results [20,21], a comprehensive prediction system for metachronous liver metastasis after radical colorectal cancer resection remains absent.

Previously, Lu et al. successfully developed a predictive model for postoperative MLM in colorectal cancer using regression analysis, achieving a C-index of 0.886 [12]. However, their study has several limitations. Specifically, the incorporated predictive variables were limited to conventional clinical parameters and lacked key biomarkers reflecting systemic inflammatory status and nutritional condition, resulting in insufficient representation of disease biological characteristics. Moreover, the sole reliance on regression analysis poses inherent limitations, as its linearity assumption fails to capture potential nonlinear relationships between biomarkers and metastatic risk [22]. Furthermore, the absence of a dedicated clinical application platform has hindered their model’s widespread implementation. In the present study, we integrated inflammatory–nutritional biomarkers with clinical parameters, employing a combined Boruta and Lasso regression feature selection strategy to systematically identify optimal predictive variables, subsequently constructing seven machine learning models for MLM prediction. Comprehensive evaluation demonstrated that the GBM model exhibited superior predictive performance (C-index = 0.916).

Patients with cancer frequently exhibit varying degrees of nutritional and metabolic disturbances. This study demonstrates that two nutritional assessment indices, the PNI and ALBI, effectively predict the risk of postoperative MLM in colorectal cancer. Notably, both indices incorporate serum albumin as a key parameter. As the primary hepatic synthetic protein, not only does serum albumin maintain plasma colloid osmotic pressure and transport functions, but its concentration variations sensitively reflect hepatic synthetic capacity and systemic immune status [23]. Tumor-induced malnutrition compromises antitumor immune surveillance by downregulating macrophage activity and inhibiting lymphocyte proliferation [24]. Consequently, in CRC, hypoalbuminemia may indicate an increased likelihood of hepatic metastasis. The lymphocyte count parameter in the PNI reflects the immunoregulatory status within the tumor microenvironment. Malignant progression exhibits complex bidirectional regulation with systemic inflammatory responses [25]. Tumor-infiltrating T lymphocytes specifically recognize tumor-associated antigens, mediating effective antitumor immune responses through cytotoxic effects and cytokine secretion [26,27]. However, malignant cells develop various immune evasion mechanisms, including the secretion of immunosuppressive factors (TGF-β, IL-10) and competitive depletion of critical cytokines (IL-2), leading to CTL exhaustion and proliferative limitation [28]. Thus, peripheral lymphocytopenia indicates both impaired antitumor immunity and tumor-mediated systemic immunosuppression [29,30]. Numerous studies have established significant associations between higher lymphocyte counts and improved therapeutic response/long-term prognosis across various malignancies [31,32]. Recent studies reveal that the PNI demonstrates prognostic significance in gastric cancer, hepatocellular carcinoma, and other malignancies [8,9]. In CRC, Li et al.’s analysis of 511 patients established 48.65 as the optimal PNI cutoff for overall prognosis prediction [11]. For postoperative MLM prediction, our study identified 44.48 as the optimal PNI cutoff. This distinction highlights that the risk of liver metastasis may be particularly sensitive to more severe degrees of nutritional–immune dysfunction than overall survival, potentially reflecting the liver’s unique vulnerability as a metastatic niche influenced by portal drainage and local immune tolerance [2,33]. Our second nutritional index, the ALBI, was originally developed to assess liver functional reserve and long-term survival in patients with HCC [34]. Although no studies directly link the ALBI to MLM, its prognostic value extends to various extrahepatic malignancies: the ALBI predicts survival outcomes (OS/PFS) in NSCLC immunotherapy [35], and shows significant mortality associations in pancreatic [36] and gallbladder cancers [20]. In these cancers, the ALBI’s albumin component reflects nutritional status, while elevated bilirubin may impair antitumor immunity by suppressing CD4+ T-cell function [37]. Furthermore, as a key liver metabolite, alterations in bilirubin levels may indirectly modulate the tumor microenvironment through influences on gut microbiota composition [38]. Bilirubin has been reported to play a role in intestinal homeostasis and host defense, and dysbiosis (e.g., decreased Bacteroides vulgatus and increased Proteus mirabilis) has been linked to suppressed Kupffer cell function in the liver and an elevated risk of hepatic metastasis [39]. Therefore, the elevated bilirubin component in the ALBI may not only directly suppress immune cell function but also contribute synergistically to fostering a metastasis-permissive microenvironment by disrupting the balance of the bilirubin–gut microbiota–liver immunity axis. Specifically, our identified ALBI threshold > −2.67 likely corresponds to bilirubin levels known to exert immunomodulatory effects and a signifies suboptimal hepatic reserve. Previously, a study investigating the relationship between the ALBI and prognosis in patients with stage III colorectal cancer identified an ALBI cutoff of −2.54 [40], but this cutoff was not suitable for predicting MLM in patients with colorectal cancer. Considering tumor-type and endpoint heterogeneity, we advocate population-specific cutoff determination. In summary, the PNI and ALBI—incorporating albumin, lymphocytes, and bilirubin—provide a comprehensive assessment of nutritional status, hepatic reserve, and immune–inflammatory balance. The specific thresholds we identified (PNI < 44.48, ALBI > −2.67) demarcate critical points where these combined deficits substantially elevate the biological propensity for hepatic colonization. These indices demonstrate significant predictive value for postoperative hepatic metastasis, showing promise as clinical biomarkers.

The SII was originally developed to evaluate systemic inflammatory responses [41]. Its clinical advantage lies in simultaneously reflecting the immune homeostasis between neutrophils and lymphocytes while incorporating the crucial role of platelets in forming the tumor metastatic microenvironment [9,10]. By integrating these three key parameters, the SII comprehensively reflects tumor-associated immune–inflammatory network dysregulation. Accumulating evidence indicates that the optimal SII cutoff is closely linked to clinical endpoints: SII = 340 stratifies high-risk subpopulations among CRC patients with identical TNM stages for overall survival prediction [42], while SII = 535 serves as an independent prognostic factor for post-hepatectomy survival in rectal cancer liver metastases [43]. However, no dedicated studies have yet reported the association between the SII and MLM development after CRC surgery. The metastasis-predictive threshold (SII > 856.36) established in this study significantly exceeds cutoffs derived from survival prognostic models. Mechanistic analysis of cancer metastasis suggests that this divergence likely reflects fundamental distinctions between two pathophysiological processes. Pro-metastatic events, characterized by rapid neutrophil extracellular trap (NET) formation and dynamic platelet–tumor cell interactions [44,45], require elevated thresholds to quantify transient activation of microenvironmental permissiveness. Conversely, chronic inflammation-induced systemic immune exhaustion (exemplified by progressive lymphocyte depletion) demonstrates greater compatibility with lower cutoffs when monitoring sustained pathological progression. Compared to conventional single inflammatory markers, the SII’s multidimensional composition may provide superior accuracy in predicting MLM risk.

A systematic review (1996–2020) incorporating multi-database analysis demonstrated that patients with fatty liver exhibit a significantly elevated risk of CRC (OR = 1.72, 95% CI: 1.40–2.11) [46]. Several studies further suggest that fatty liver disease may facilitate CRC liver metastasis, potentially through the upregulation of fatty acid synthase (FASN) in the lipid-rich microenvironment, enhancing palmitate synthesis in CRC cells and thereby promoting hepatic metastatic potential [47,48]. Interestingly, one published study reported contradictory findings, suggesting that fatty liver disease in patients with CRC may reduce hepatic metastasis risk [49]. Similarly, Wu et al. observed in a retrospective study that breast cancer patients with concurrent fatty liver disease experienced significantly earlier liver metastasis compared to those without fatty liver (*p* = 0.022) [50]. Our SHAP analysis also aligns with this protective effect hypothesis. We suggest that although metabolic disturbances in fatty liver disease—such as insulin resistance and chronic inflammation—may promote tumorigenesis [51], the metastatic process exhibits distinct biological constraints, with only a minority of tumor cells successfully overcoming vascular barriers and microenvironmental challenges to establish distant organ colonization [52]. Additionally, fatty liver may create an anti-metastatic microenvironment by suppressing angiogenesis and reducing thymidine phosphorylase activity [53]. However, these findings should not be misconstrued as supporting a high-fat diet. A scientifically balanced diet and healthy eating habits remain fundamental to maintaining overall health.

In addition to the novel inflammatory–nutritional biomarkers described above, this study further validates the predictive value of established clinical parameters. Carcinoembryonic antigen (CEA), a widely utilized tumor marker in clinical practice, demonstrates significant diagnostic value for early malignancy detection when abnormally elevated [54]. Our investigation revealed a notable nonlinear relationship between CEA levels and MLM prediction. While CEA > 5 μg/L is conventionally employed as a malignancy screening threshold, our findings suggest that higher CEA levels (>8.14 μg/L) may be more appropriate for metastatic risk stratification. This elevated threshold may better reflect a tumor burden or biological aggressiveness sufficient to seed successful liver metastases. It potentially demarcates a transition point where CEA’s role extends beyond mere tumor presence to actively promoting metastatic processes. The TNM staging system established by the American Joint Committee on Cancer (AJCC) has gained universal acceptance for CRC clinical staging [55]. Studies confirm that tumor progression to T3–4 stage (deep muscularis propria invasion) and N1–2 stage (lymph node metastasis) significantly increases the risk of tumor cell dissemination via the portal venous system due to compromised anatomical barriers. This finding has been substantiated in previous research [12]. Notably, some studies demonstrate that distant metastases may still occur in node-negative patients [56], necessitating multidimensional evaluation incorporating additional variables. In this study, we integrated inflammatory–nutritional markers with clinical and pathological parameters into CRC risk stratification, resulting in a comprehensive prediction model with satisfactory performance.

This study has several inherent limitations. First, while our missing data imputation strategy (mean/mode for variables with <5% missingness) was appropriate given the low proportion of missing values, we acknowledge several implications: mean imputation may reduce variance in continuous variables, leading to underestimated standard errors, and mode imputation could slightly inflate the frequency of the most common category; this may consequently cause potential slight distortions in model parameter estimation and subsequent statistical inference. Second, given that this was a single-center retrospective study, although consecutive case enrollment was adopted to minimize selection bias, the sample representativeness may still be constrained by the unique clinical characteristics of a single institution. More critically, while internal validation through rigorous split-cohort methods was performed, the lack of external validation remains a major constraint. Our model’s performance may be influenced by institution-specific practices (e.g., surgical techniques, biomarker assays) and demographic biases, limiting its generalizability to other populations. Third, beyond data-related limitations, the insufficient standardization of inflammatory–nutritional biomarker assays, coupled with the lack of real-time data integration between the prediction platform and hospital information systems, presents challenges for clinical implementation. To address these limitations, we have initiated a multicenter collaboration across three geographic regions (East China, North China, and Southwest China) to validate the model prospectively. This initiative will enroll > 2000 patients with CRC with standardized protocols for biomarker measurement and follow-up schedules. Additionally, we plan to establish an open-access web platform to allow external users to test the model with local data, further evaluating its adaptability across diverse healthcare settings. Future studies should also explore the incorporation of molecular markers and radiomic features to enhance predictive accuracy.

## 5. Conclusions

Machine learning models serve as robust tools for predicting the risk of MLM. The GBM model, incorporating clinicopathological features and inflammatory–nutritional biomarkers, demonstrated superior predictive performance, with Shapley values and generalized additive models elucidating critical thresholds and nonlinear patterns of CEA, the PNI, the SII, and the ALBI. These findings provide quantitative evidence for formulating personalized surveillance strategies, while the developed dynamic prediction tool facilitates clinical translation of these research outcomes.

## Figures and Tables

**Table 1:** Clinical characteristics of patients in the total, training, and testing cohorts.

Characteristics	Total Cohort	Training Cohort	Testing Cohort
	n = 680	n = 477	n = 203
Gender			
Male	322 (47.4)	235 (49.3)	87 (42.9)
Female	358 (52.6)	242 (50.7)	116 (57.1)
Diabetes			
Yes	116 (17.1)	85 (17.8)	31 (15.3)
No	564 (82.9)	392 (82.2)	172 (84.7)
Fatty liver			
Yes	183 (26.9)	137 (28.7)	46 (22.7)
No	497 (73.1)	340 (71.3)	157 (77.3)
T stage			
T1–2	197 (29.0)	145 (30.4)	52 (25.6)
T3–4	483 (71.0)	332 (69.6)	151 (74.4)
N stage			
N0	344 (50.6)	253 (53.0)	91 (44.8)
N1–2	336 (49.4)	224 (47.0)	112 (55.2)
Differentiation grade			
G1–2	521 (76.6)	366 (76.7)	155 (76.4)
G3–4	159 (23.4)	111 (23.3)	48 (23.6)
Colonic obstruction			
Yes	106 (15.6)	76 (15.9)	30 (14.8)
No	574 (84.4)	401 (84.1)	173 (85.2)
Vascular invasion			
Yes	291 (42.8)	202 (42.3)	89 (43.8)
No	389 (57.2)	275 (57.7)	114 (56.2)
Perineural invasion			
Yes	166 (24.4)	119 (24.9)	47 (23.2)
No	514 (75.6)	358 (75.1)	156 (76.8)
Maximal size of regional lymph node (%)			
<5 mm	274 (40.3)	195 (40.9)	79 (38.9)
5–10 mm	301 (44.3)	216 (45.3)	85 (41.9)
>10 mm	105 (15.4)	66 (13.8)	39 (19.2)
mGPS (%)			
0	275 (40.4)	184 (38.6)	91 (44.8)
1	269 (39.6)	192 (40.3)	77 (37.9)
2	136 (20.0)	101 (21.2)	35 (17.2)
Age	66.53 (9.59)	66.51 (9.13)	66.60 (10.61)
BMI	22.53 (3.39)	22.47 (3.35)	22.68 (3.48)
AFP	3.09 (1.92)	3.16 (1.97)	2.92 (1.78)
CA125	44.39 (104.96)	41.89 (99.86)	50.27 (116.13)
CA199	34.96 (93.03)	36.79 (95.70)	30.66 (86.51)
CEA	7.85 (7.18)	7.67 (6.90)	8.26 (7.80)
Operation time	208.98 (88.98)	210.39 (88.76)	205.65 (89.62)
Blood loss	267.01 (294.69)	265.53 (291.84)	270.50 (302.00)
SIRI	1.55 (1.75)	1.57 (1.76)	1.50 (1.72)
PNI	43.61 (6.97)	43.44 (6.98)	44.03 (6.94)
SII	825.19 (630.17)	840.36 (639.21)	789.55 (608.47)
ALBI	−2.65 (0.24)	−2.65 (0.23)	−2.63 (0.26)
Tumor size	3.62 (2.03)	3.66 (2.00)	3.52 (2.11)

**Table 2:** Clinical characteristics of patients with CRC and MLM in the training cohort.

Characteristics	Training Cohort			*p* Overall
	Overall n = 477	CRC n = 290	MLM n = 187	
Gender				0.39
Male	235 (49.3)	148 (51.0)	87 (46.5)	
Female	242 (50.7)	142 (49.0)	100 (53.5)	
Diabetes				0.59
Yes	85 (17.8)	49 (16.9)	36 (19.3)	
No	392 (82.2)	241 (83.1)	151 (80.7)	
Fatty liver				<0.001
Yes	137 (28.7)	107 (36.9)	30 (16.0)	
No	340 (71.3)	183 (63.1)	157 (84.0)	
T stage				<0.001
T1–2	145 (30.4)	107 (36.9)	38 (20.3)	
T3–4	332 (69.6)	183 (63.1)	149 (79.7)	
N stage				<0.001
N0	253 (53.0)	198 (68.3)	55 (29.4)	
N1–2	224 (47.0)	92 (31.7)	132 (70.6)	
Differentiation grade				<0.001
G1–2	366 (76.7)	256 (88.3)	110 (58.8)	
G3–4	111 (23.3)	34 (11.7)	77 (41.2)	
Colonic obstruction				0.23
Yes	76 (15.9)	41 (14.1)	35 (18.7)	
No	401 (84.1)	249 (85.9)	152 (81.3)	
Vascular invasion				<0.001
Yes	202 (42.3)	79 (27.2)	123 (65.8)	
No	275 (57.7)	211 (72.8)	64 (34.2)	
Perineural invasion				0.4
Yes	119 (24.9)	68 (23.4)	51 (27.3)	
No	358 (75.1)	222 (76.6)	136 (72.7)	
Maximal size of regional lymph node (%)				0.11
<5 mm	195 (40.9)	126 (43.4)	69 (36.9)	
5–10 mm	216 (45.3)	131 (45.2)	85 (45.5)	
>10 mm	66 (13.8)	33 (11.4)	33 (17.6)	
mGPS (%)				0.01
0	184 (38.6)	112 (38.6)	72 (38.5)	
1	192 (40.3)	129 (44.5)	63 (33.7)	
2	101 (21.2)	49 (16.9)	52 (27.8)	
Age	66.51 (9.13)	66.16 (9.14)	67.04 (9.12)	0.31
BMI	22.47 (3.35)	22.22 (3.21)	22.87 (3.54)	0.04
AFP	3.16 (1.97)	3.13 (1.89)	3.20 (2.09)	0.67
CA125	41.89 (99.86)	48.70 (108.94)	31.33 (83.03)	0.06
CA199	36.79 (95.70)	39.41 (102.85)	32.74 (83.51)	0.46
CEA	7.67 (6.90)	5.93 (5.60)	10.37 (7.83)	<0.001
Operation time	210.39 (88.76)	211.82 (89.63)	208.18 (87.57)	0.66
Blood loss	265.53 (291.84)	274.03 (312.51)	252.34 (256.76)	0.43
SIRI	1.57 (1.76)	1.54 (1.79)	1.61 (1.70)	0.68
PNI	43.44 (6.98)	44.30 (7.18)	42.09 (6.46)	0.001
SII	840.36 (639.21)	672.90 (433.92)	1100.05 (800.97)	<0.001
ALBI	−2.65 (0.23)	−2.71 (0.20)	−2.57 (0.25)	<0.001
Tumor size	3.66 (2.00)	3.68 (2.05)	3.62 (1.91)	0.76

**Table 3:** Comprehensive performance assessment of seven models in testing cohort.

Model	AUROC (95% CI)	Accuracy (95% CI)	Sensitivity (95% CI)	Specificity (95% CI)	Precision (95% CI)	F1 (95% CI)
GBM	0.916 [0.879–0.952]	0.833 [0.781–0.884]	0.772 [0.680–0.865]	0.871 [0.812–0.930]	0.792 [0.703–0.882]	0.782 [0.691–0.873]
Logistic	0.884 [0.839–0.931]	0.803 [0.748–0.858]	0.861 [0.784–0.937]	0.766 [0.692–0.841]	0.701 [0.600–0.802]	0.773 [0.680–0.865]
SVM	0.882 [0.836–0.928]	0.808 [0.754–0.862]	0.823 [0.739–0.907]	0.798 [0.728–0.869]	0.722 [0.623–0.821]	0.769 [0.676–0.862]
Neural Network	0.883 [0.837–0.928]	0.793 [0.737–0.849]	0.797 [0.709–0.886]	0.79 [0.719–0.862]	0.708 [0.608–0.808]	0.75 [0.655–0.845]
KNN	0.864 [0.812–0.916]	0.803 [0.748–0.858]	0.899 [0.832–0.965]	0.742 [0.665–0.819]	0.689 [0.587–0.791]	0.78 [0.689–0.872]
AdaBoost	0.701 [0.632–0.769]	0.7 [0.636–0.763]	0.684 [0.581–0.786]	0.71 [0.630–0.790]	0.6 [0.492–0.708]	0.639 [0.533–0.745]
CatBoost	0.882 [0.836–0.927]	0.813 [0.759–0.866]	0.734 [0.637–0.832]	0.863 [0.802–0.923]	0.773 [0.681–0.866]	0.753 [0.658–0.848]

## Data Availability

The original contributions presented in this study are included in the article/Supplementary Materials. Further inquiries can be directed to the corresponding authors.

## Supplementary Materials

The following supporting information can be downloaded at https://www.mdpi.com/article/10.3390/biomedicines13071706/s1, Figure S1: (A) Bar plot showing the percentage of missing values for each of the 24 features. (B) Red squares represent missing values; Table S1: Parameters of the Seven Models; Table S2: Comprehensive performance assessment of seven models in training cohort.
