# Supplementary material for: An Interpretable Machine Learning Model Based on Inflammatory–Nutritional Biomarkers for Predicting Metachronous Liver Metastases After Colorectal Cancer Surgery
Source: Biomedicines. 2025 Jul 12;13(7):1706. doi: 10.3390/biomedicines13071706 (PMC12292804; doi:10.3390/biomedicines13071706)

Figure.S1 (A) Bar plot showing the percentage of missing values for each of the 24 features. **(B)** Red squares represent missing values.

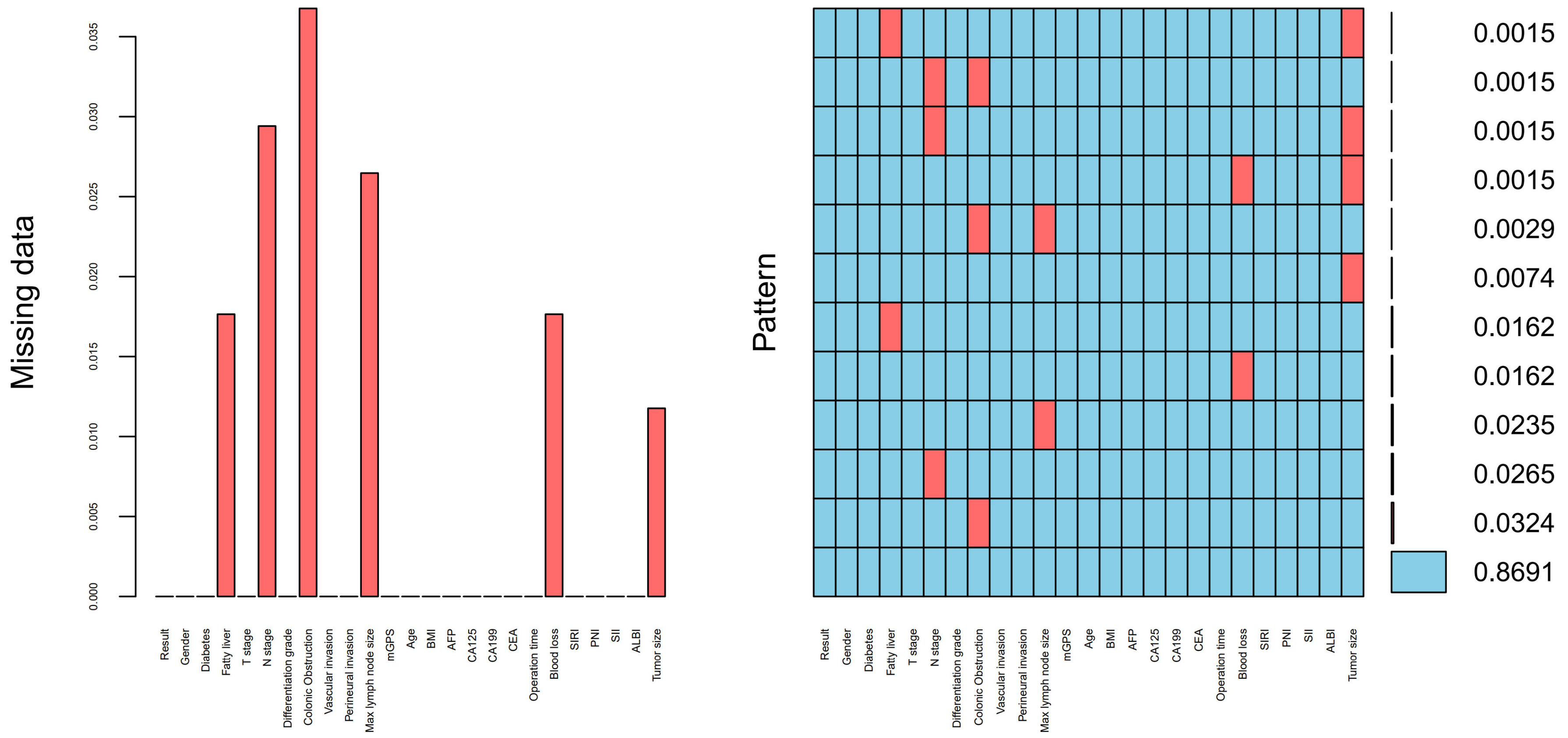

Supplement: Supplementary file 1 [file biomedicines-13-01706-s001.zip › Figure S1.pdf]
