# Supplementary material for: An Interpretable Machine Learning Model Based on Inflammatory–Nutritional Biomarkers for Predicting Metachronous Liver Metastases After Colorectal Cancer Surgery
Source: Biomedicines. 2025 Jul 12;13(7):1706. doi: 10.3390/biomedicines13071706 (PMC12292804; doi:10.3390/biomedicines13071706)
Supplement: Supplementary file 1 [file biomedicines-13-01706-s001.zip › Table S1.pdf]

Table S1. Parameters of the Seven Models

**Algorithm**	**Optimal Parameter**
**LR**	C=100, penalty=l1, solver=liblinear
**SVM**	sigma = 0.001, C = 0.3
**GBM**	n.trees=100,interaction.depth=3,shrinkage = 0.1, n.minobsinnode = 5
**Neural networks**	size = 4,decay = 0.6
**KNN**	k=12, diatance=1, kernel = "optimal"
**AdaBoost**	mfinal = 2,maxdepth = 2
**CatBoost**	border_count = 32,depth = 5, learning_rate = 0.03

LR, Logistic Regression; SVM, Support Vector Machine; GBM, gradient boosting machine; KNN, K-nearest neighbors;
