# Supplementary material for: An Interpretable Machine Learning Model Based on Inflammatory–Nutritional Biomarkers for Predicting Metachronous Liver Metastases After Colorectal Cancer Surgery
Source: Biomedicines. 2025 Jul 12;13(7):1706. doi: 10.3390/biomedicines13071706 (PMC12292804; doi:10.3390/biomedicines13071706)
Supplement: Supplementary file 1 [file biomedicines-13-01706-s001.zip › Table S2.pdf]

Table S2. Comprehensive performance assessment of seven models in training cohort

Model	AUROC (95% CI)	Accuracy (95% CI)	Sensitivity (95% CI)	Specificity (95% CI)	Precision (95% CI)	F1 (95% CI)
GBM	0.966 [0.952-0.980]	0.887 [0.858-0.915]	0.968 [0.943-0.993]	0.834 [0.792-0.877]	0.79 [0.732-0.849]	0.87 [0.822-0.918]
Logistic	0.905 [0.878-0.932]	0.841 [0.808-0.874]	0.866 [0.818-0.915]	0.824 [0.780-0.868]	0.761 [0.699-0.822]	0.81 [0.754-0.866]
SVM	0.904 [0.877-0.931]	0.836 [0.803-0.870]	0.872 [0.824-0.920]	0.814 [0.769-0.859]	0.751 [0.689-0.813]	0.807 [0.750-0.864]
NeuralNetwork	0.912 [0.887-0.938]	0.839 [0.806-0.872]	0.872 [0.824-0.920]	0.817 [0.773-0.862]	0.755 [0.693-0.816]	0.809 [0.753-0.865]
KNN	0.956 [0.941-0.971]	0.872 [0.842-0.902]	0.957 [0.928-0.986]	0.817 [0.773-0.862]	0.772 [0.711-0.832]	0.854 [0.804-0.905]
Adaboost	0.759 [0.718-0.800]	0.755 [0.716-0.793]	0.706 [0.641-0.771]	0.786 [0.739-0.833]	0.68 [0.614-0.747]	0.693 [0.627-0.759]
CatBoost	0.933 [0.911-0.955]	0.853 [0.821-0.885]	0.893 [0.849-0.937]	0.828 [0.784-0.871]	0.77 [0.709-0.830]	0.827 [0.772-0.881]
